# The role of V-shaped oceans and ribbon continents in the Brasiliano/PanAfrican assembly of western Gondwana

**DOI:** 10.1038/s41598-023-28717-7

**Published:** 2023-01-28

**Authors:** Fabrício de Andrade Caxito, Fernando Flecha Alkmim

**Affiliations:** 1 CPMTC Research Center and Departamento de Geologia, Universidade Federal de Minas Gerais, Belo Horizonte, MG 31270‑901 Brazil; 2 Departamento de Geologia, Escola de Minas, Universidade Federal de Ouro Preto, Ouro Preto, MG 35400-000 Brazil

**Keywords:** Tectonics, Precambrian geology

## Abstract

Western Gondwana amalgamated by collision of continental blocks that did not form prior conjugated margins (extroversion), and by typical Wilson cycles, when continental blocks that rifted away giving birth to new oceans were subsequently re-joined in approximately the same position (introversion). The introverted systems are characterized by the opening of V-shaped basins through rifting and hyperextension of various continental pieces (micro- and ribbon continents) from a former Central African Block. These continental fragments lost substantial parts of their mantle lithosphere and became decratonized while drifting towards the external Goiás-Pharusian ocean. Protracted seafloor spreading and consumption through subduction of the internal and external oceans, respectively, ultimately led to multiple, diachronous collisions with other continental blocks detached from Rodinia (Amazonian, West Africa, Embu, etc.). These collisions pushed the ribbon continents back and closed the introverted basins, squeezing and incorporating the reworked basement tracts between the main colliding blocks and the rigid remainder of the Central African Block (the São Francisco-Congo craton). Continental extrusion and lateral escape tectonics ensued, generating thousands-of-km long networks of anastomosing directional shear zones (keirogens), as a consequence of both the accretionary systems developed between the involved blocks and the highly deformable nature of the decratonized ribbon continents.

## Introduction

**Figure 1:**
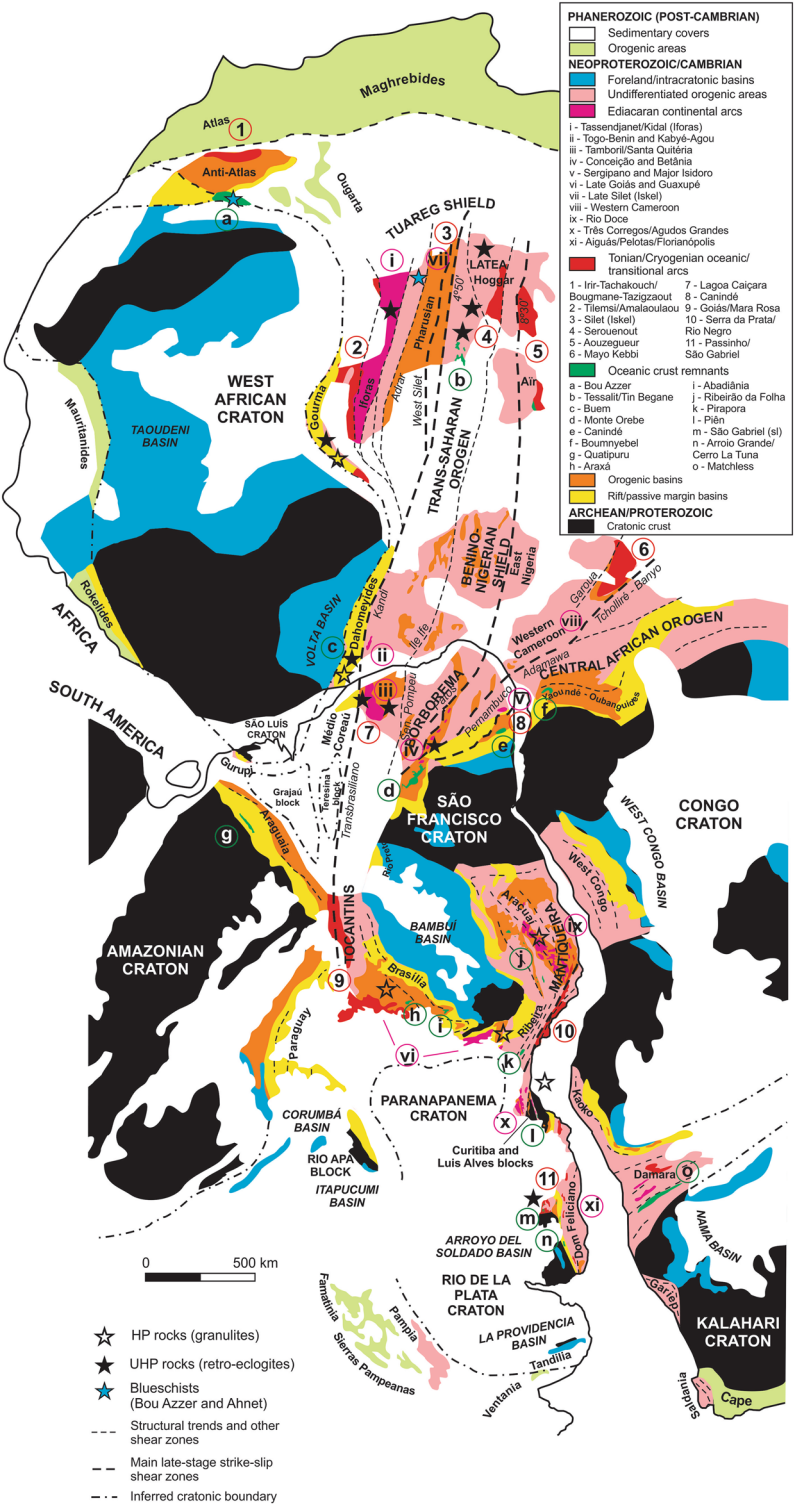
Simplified geology of eastern South America and western Africa in a pre-drift configuration. Dashed lines represent main shear zones. Map created using Corel Draw Graphics Suite 2018 (http://www.coreldraw.com) and a Huion Kamvas GS1331B pen display (http://www.huion.com).

The western portion of Gondwana, now represented by South America and Africa (Fig. 1), is composed of a mosaic of Archean/Paleoproterozoic cratons and Neoproterozoic to Cambrian Brasiliano/PanAfrican orogenic systems. Evolutionary models postulated for this region address three main issues, which from time to time are revisited and intensively debated: (i) nature of the Brasiliano/PanAfrican orogenic systems, i.e. whether they formed through oceanic lithosphere consumption and collision, as a result of mainly intracontinental processes, or as a combination of both; (ii) genealogy of the continental masses involved, which are represented by cratons and crustal blocks of variable sizes, i.e. if they were or not part of specific ancient supercontinents such as Rodinia and Columbia; (iii) the sequence in which the assembly took place (see, among others^1–5^).

The cratons of South America and Africa (Fig. 1) correspond to lithospheric pieces that largely escaped the thick-skinned effects of the Brasiliano/PanAfrican orogenies^6^. They consist essentially of Archean and Proterozoic crust locally endowed with thick lithospheric roots (e.g.^7–13^) and partially covered by Precambrian and Phanerozoic (meta-)volcanosedimentary successions. Most of them attained final stabilization around 1.0 Ga and their sedimentary covers include Tonian-Cryogenian rift sequences, marginal Cryogenian-Ediacaran passive-margin deposits, and Ediacaran-Cambrian foreland basins (e.g.^14–16^).

The Brasiliano/PanAfrican systems form a network of orogens developed between the South American and African cratons (Fig. 1) mainly in the time interval of 630 to 500 Ma. The external fold-thrust belts of these systems incorporate the reworked margins of the cratons, composed of basement (older than 1.8 Ga) partially covered by pre-Tonian sedimentary units, Tonian to early Ediacaran pre-rift, rift, and passive margin successions, as well as foreland basins.

The internal portions of the orogenic systems, on the other hand, consist of 880–620 Ma island arc assemblages, 680–590 Ma continental arc rocks, ophiolitic and syn-orogenic sedimentary units, syn- to post-collisional granites, and reworked basement blocks (older than 1.8 Ga, commonly referred to as inliers, micro-continents or median massifs^17^). For simplicity, the systems focused on this paper will be hereafter referred to as the Tocantins (Central Brazil), Borborema/NW Africa (including the Dahomeyides, Trans-Saharan, and Oubanguides), and Mantiqueira/SW Africa (encompassing the West Congolian, Kaoko, Gariep and Saldania belts) (Fig. 1), following the classic nomenclature^6^.

Attempts to reconstruct the geodynamic evolution of western Gondwana were hampered by two aspects of the Brasiliano/PanAfrican systems: (i) the presence, in the Neoproterozoic/Cambrian orogenic systems, of large tracts dominated by reworked Archean-Paleoproterozoic basement; and (ii) obliteration of previous fabric elements by thousands-of-km long strike-slip systems developed after the peak of collisional metamorphism and magmatism. Here we propose a model for the evolution of western Gondwana that reconciles these factors and its main traits as described above. The keystone of our model is the disaggregation of a Proterozoic continent, the Central African Block^18–20^, which encompassed both the São Francisco-Congo cratonic nuclei and most of the continental fragments that are now recognized as reworked basement tracts within the surrounding Brasiliano/PanAfrican domains. We discuss the evidence favoring the persistence of the Central African continent during much of the Proterozoic Eon and the role played by the fragments derived from it in the assembly and final configuration of western Gondwana.

## Putative “Ariadne’s threads” for the reconstruction of the Central African block

**Figure 2:**
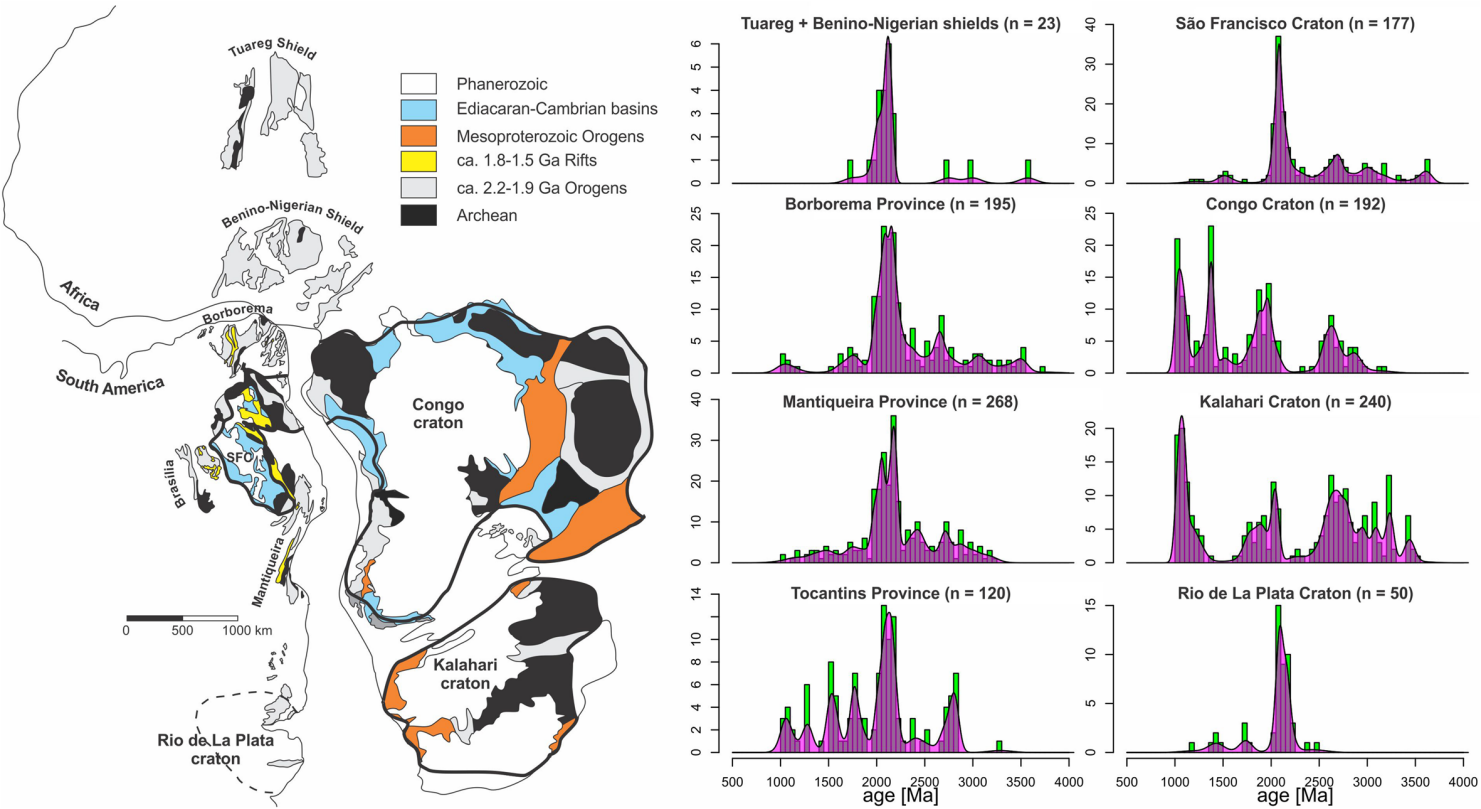
Putative Ariadne’s threads for the existence of the Proterozoic Central African Block^18^. Igneous zircon distributions (only samples older than 1.0 Ga included) are compiled from the open sources DateView^74^ and the Geological Survey of Brazil database (http://geosgb.cprm.gov.br), and histograms and probability density plots were prepared through IsoPlotR^75^. Note the occurrence of an important ca. 2.2–1.9 Ga crust forming event in all of the studied provinces, followed in size by a 2.5–2.7 Ga peak. Map and drawing created using Corel Draw Graphics Suite 2018 (http://www.coreldraw.com) and a Huion Kamvas GS1331B pen display (http://www.huion.com).

Regarding the genealogy of the South American and African cratons, most authors agree on their derivation from Rodinia^21^, except for the case of the São Francisco-Congo craton (Fig. 1). Paleocontinental reconstructions for 1.0 Ga portray the latter either as an isolated landmass^22–27^ or in the periphery of Rodinia^28–30^. A different view of the Mesoproterozoic paleocontinental scenario was recently put forward^18–20^. Accordingly, the São Francisco-Congo and the non-cratonic Borborema-Transaharan orogenic system (Fig. 1) once formed a single lithospheric entity, the Central African block^18^ (Fig. 2), which might also have incorporated parts of the basement tracts now preserved in the Tocantins and Mantiqueira provinces^20^, and possibly even the Kalahari and Rio de La Plata cratons^18–20^. This landmass remained assembled from around 2.0 Ga until ca. 0.9 Ga, when a substantial part of its components were rifted from its margins and drifted away^5,19,20,31^. Despite the paucity of paleomagnetic control, this hypothesis finds support in a series of common features shared by the majority of the mentioned cratonic and non-cratonic domains. These are:

1. The presence of Archean seed nuclei as old as ca. 3.64–3.00 Ga in both the cratonic area^32,33^ and in the reworked basement of the Borborema/NW Africa^34–38^, Tocantins^39^ and Mantiqueira/SW Africa^40,41^ provinces.
2. Major 2.7–2.5 Ga greenstone belts and plutonic rocks surrounding, intruding and reworking the Archean seed nuclei, formerly known as “Jequié” or Rio das Velhas cycle in the cratonic area^33,42^, also recognized in the Borborema/NW Africa^35,43–46^, Tocantins^47^ and in the Mantiqueira/SW Africa^40,41^ provinces.
3. The occurrence of Siderian-Rhyacian basins (ca. 2.5–2.2 Ga) filled with rift to passive margin successions, covered by syn- to post-orogenic (foreland) successions, as well as volcanosedimentary units and plutonic assemblages partially affected by the 2.1–2.0 Ga Transamazonian/Eburnean orogeny in all of the basement domains. The Siderian-Rhyacian basins configure a coherent paleogeographic picture when restored into western Gondwana (Fig. 2) and are materialized by the following successions: the Francevillian Group in the Congo craton in Gabon^48–50^; the Kimezian Group, intensively affected by the PanAfrican orogeny along the coast of Angola^51,52^, apparently representing the extension of the former further south along the Congo cratonic margin; the Minas Supergroup and the Itacolomi Group exposed in the Quadrilátero Ferrífero mineral province in the southern border of the São Francisco craton in Brazil^16,53,54^; the Transvaal Supergroup in the western Kalahari craton, Griqualand West, South Africa^55,56^; and similarly aged metavolcanosedimentary successions composing the basement of the Mantiqueira/SW Africa, Tocantins and Borborema/NW Africa provinces.
4. Regionally extensive Rhyacian-Orosirian orogenic belts with mean peak of activity in the 2.2–1.9 Ga age range. This is the so-called “Transamazonian” or Minas-Bahia Orogeny of the Mineiro and Itabuna-Salvador-Curaçá belts within the São Francisco craton domain^42,57,58^, and is indeed the main episode of crust formation in the São Francisco-Congo, Borborema/NW Africa, Tocantins and Mantiqueira/SW Africa provinces, readily recognizable in igneous zircon U–Pb age distribution plots (Fig. 2). Arguably, this orogenic cycle was responsible for the consolidation of the Central African Block.
5. The presence of widespread, synchronous and well-developed continental rift systems during the Paleo-Mesoproterozoic in both the São Francisco-Congo craton^59,60^ and the basements of the Borborema/NW Africa^61,62^, Tocantins^63^ and Mantiqueira/SW Africa^64–66^ provinces, which attest for contiguity on a former single large continental landmass. Continental rift bimodal volcanic and plutonic units peaks at 1.8–1.7 Ga and 1.6–1.5 Ga, also marked by regional dyke swarms in the same age ranges^20,67^, followed by continental to shallow marine sag basins.
6. Continental rifting in the Early Tonian, at 900–850 Ma, marked by widespread regional mafic dyke swarms^20,30^. Unlike the Paleo-Mesoproterozoic rifting events, the early Tonian episode eventually led to continental breakup and opening of new oceanic basins. In effect, the margins of the São Francisco-Congo paleocontinent are rimmed by mafic-ultramafic^68^ and A-type granite^69^ intrusions in the Borborema Province^68^, A-type granites in the northern Mantiqueira Province^70^, and thick bimodal volcanic successions especially developed in the West Congo Belt, the African counterpart of the northern end of the Mantiqueira Province^51,71^. Likewise, the Apiaí gabbro in the southern Mantiqueira^72^ was intruded at 877 Ma. Those units testify for the emplacement of a mantle plume (generating the Bahia-Gangila LIP^73^) and crustal thinning, which started to shape the São Francisco-Congo paleocontinent in its known form through peeling and detachment of continental fragments from its margins.

Taken together, the evidence above supports the hypothesis that the São Francisco-Congo craton and the reworked basement in the Mantiqueira/SW Africa, Tocantins, and Borborema/NW Africa might once have been part of a single landmass. This landmass involved Archean seed nuclei amalgamated by an extensive Paleoproterozoic orogenic network (2.2–1.9 Ga), which then underwent multiple attempts to breaking up at ca. 1.8–1.7 Ga, 1.6–1.5 Ga, probably ca.1.2 Ga, and eventually at 900–850 Ma. These similarities were previously noted by other authors, especially for the Borborema Province in comparison to the São Francisco craton^31,76^. Some authors argue in favor of evolution of the Brasiliano/PanAfrican systems from closure of mainly large scale intracontinental rift systems that would have developed within this continuous basement^76,77^. However, according to our view, during the Tonian and Cryogenian extensional events, rifting of substantial portions of the Central African Block led to the development of V-shaped gulfs partially floored by oceanic crust, as well as micro- and ribbon continents that would drift relatively freely as distinct plates, and eventually reunite to form the Mantiqueira/SW Africa and Borborema/NW Africa systems. Birth and consumption of these ancient oceans are recorded by scraps of Neoproterozoic oceanic lithosphere enclosed in accretionary mélanges^78–80^, island- and continental-margin arc systems^81–83^ and other plate tectonics markers preserved between the major reworked ribbon continents.

## Hyperextension of the Central African block and formation of ribbon continents

Various names have been used to describe areas of reworked Archean/Paleoproterozic basement within the Brasiliano orogenic systems^17^, such as “median massifs”, “inliers”, “marginal massifs” or simply “blocks”. Reworked basement in these cases might correspond to: (i) large basement highs within rifted continental margins (such as the Guanhães and Porteirinha blocks representing the reworked margins of the São Francisco craton in the northern Mantiqueira system and the Almas-Dianópolis block in the Tocantins system^17^); (ii) allochtonous microcontinents and other exotic terranes that become entrained in orogenic belts during collision (such as the Embu terrane in the Mantiqueira system^84,85^); (iii) micro- and ribbon continents and other continental blocks generated by hyperextension and detached from former continental margins through the opening of new oceanic basins (such as the Borborema ribbon continents^5,31^). Continental fragments subject to hyperextension can lose part of their mantle roots, becoming thus susceptible to contractional and strike-slip deformation during further episodes of collision and lateral escape tectonics. In other words, these fragments become decratonized, such as in today’s eastern part of the North China craton^86^. In various segments of the Brasiliano/PanAfrican systems, these fragments of reworked continental lithosphere are surrounded by orogenic regions containing ophiolites, retro-eclogites, pre-, syn- and post-collisional magmatism, deformation, and metamorphism, indicating the operation of Phanerozoic-style plate tectonics processes (Fig. 1). Thus, orogenic reworking of older basement in areas characterized by these components should not be confused with the concept of intracontinental orogeny^87^, where none of the above cited tectonic markers can be found.

**Figure 3:**
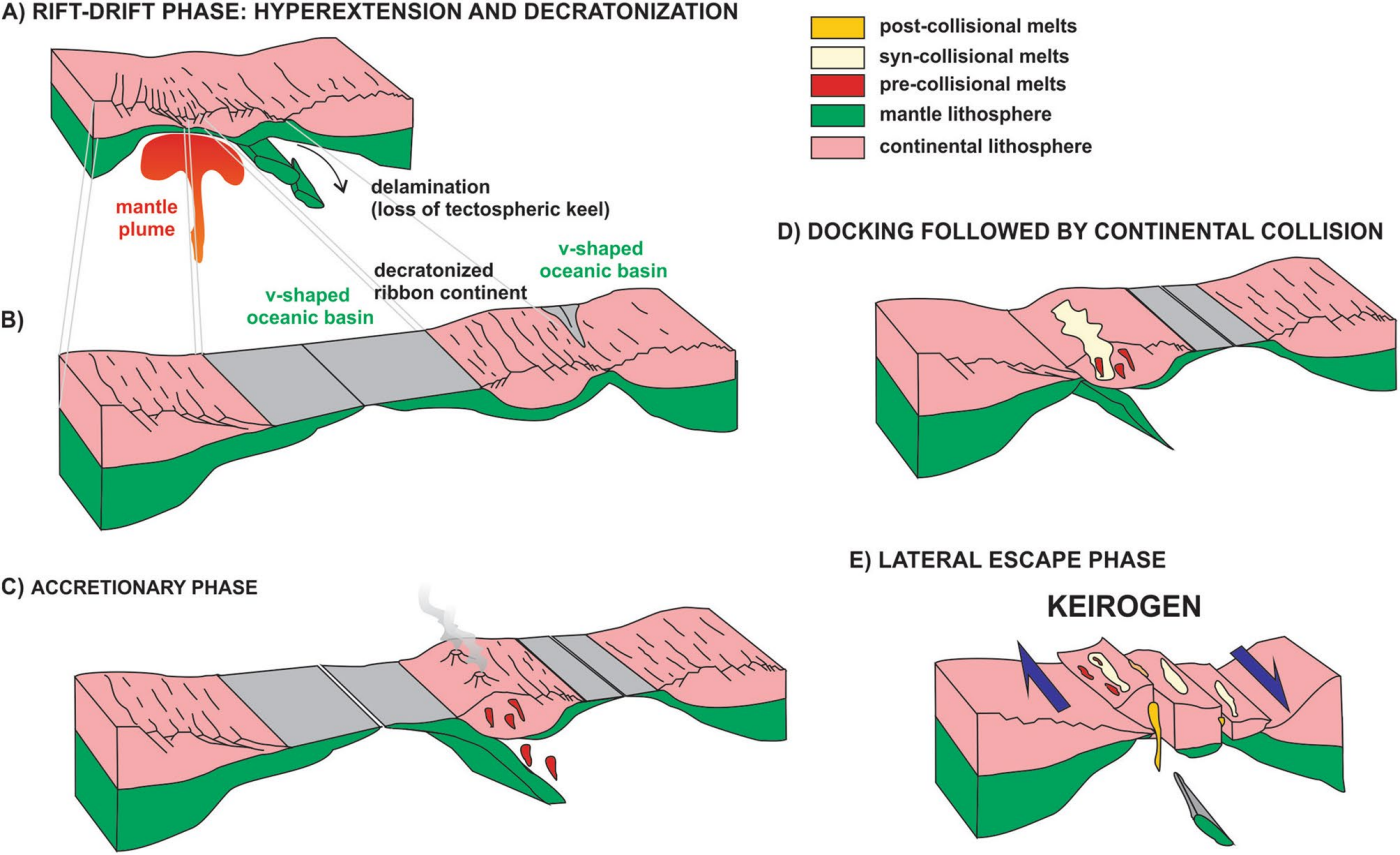
Formation of ribbon continents through hyperextension and loss of the tectospheric keel (**A**) leading to decratonization (**B**) of marginal continental tracts. Upon subduction (**C**), accretion and collision (**D**), the decratonized ribbon continents are more easily deformable, metamorphosed and injected by pre- (C), syn- (D) and post-collisional (**E**) plutons. If contractional deformation is not able to accommodate stresses in continental collision, lateral escape tectonics ensues, with formation of continental-scale shear systems (keirogens^88^). Drawing created using Corel Draw Graphics Suite 2018 (http://www.coreldraw.com) and a Huion Kamvas GS1331B pen display (http://www.huion.com).

We argue that large-scale extensional processes related to the emplacement of the ca. 900–850 Ma plume heads of the Bahia-Gangila LIP^5,31,68,73^ initiated the disaggregation of the Central African Block into micro- and ribbon continents (Fig. 3), delineating thus the São Francisco-Congo cratonic core as the only rigid relic of a former much larger paleocontinent. Furthermore, this process led to the opening of various V-shaped oceanic basins that separated the rifted micro- and ribbon continents from the main paleocontinental mass, characterizing the Transnordestino-Central African and Adamastor oceanic realms (Fig. 3). These were later converted, respectively, into the Borborema-NW Africa and Mantiqueira-SW Africa systems during the Brasiliano/PanAfrican orogenies.

Ribbon continents are long and narrow strips of continental lithosphere that become detached from a continental margin during continental extension^89–91^. They can remain relatively unthinned (> 20 km thick crust^91^) and bounded either by oceanic crust or inland rift systems. Some authors recommend to reserve the denomination to pieces of land that are still punctually connected to the unstretched continental margin in proximal settings, and use the term “microcontinents” to continental blocks fully surrounded by oceanic crust^91^. Other authors^90^ suggest the use of the term for both the continental tracts fully detached from the main continental mass, and the segments that have only one “free end”, i.e., that are still punctually connected to the mainland. In both cases, the importance of V-shaped basins in separating various types of extensional blocks of continental lithosphere in rifted margins is recognized^91^.

Ribbon continents are recognized in various ancient orogenic systems throughout the world, from the Archean to the Cenozoic^89,90,92–95^. Conversely, rifting, dispersion and accretion of micro- and ribbon continents from one paleocontinental mass to another in a “windshield-wiper fashion”^90^ seem to have been involved in the opening and closure of various ancient oceans. Rifting of continental blocks such as Avalonia-Cadomia from the margins of Gondwana, for example, gave rise to the Rheic ocean^96^, which expanded while the older Iapetus ocean shrank due to drifting of those blocks and ultimately closed upon their collision with Laurentia. Similarly, the Neotethys expanded while the Paleotethys was being consumed, separated by ribbon continents such as Cimmeria and Sibumasu^93,97–99^. The Mongol-Okhotskh ocean was closed due to rifting and dispersion of Amuria from Siberia^100^, and Paleo-Pacific basins were terminated by accretion of the SAYBIA ribbon continent to the Northwestern American margin^92^. In western Gondwana itself, the Damara belt was recently interpreted as the result of opening and closure of two basins (the northern Outjo, opened ~ 655 − 645 Ma and closed ~ 610 − 600 Ma, and the southern Khomas, opened ~ 635 Ma(?) and closed ~ 550 Ma), separated by a ribbon continent (Central or Swakop terrane)^101^.

**Figure 4:**
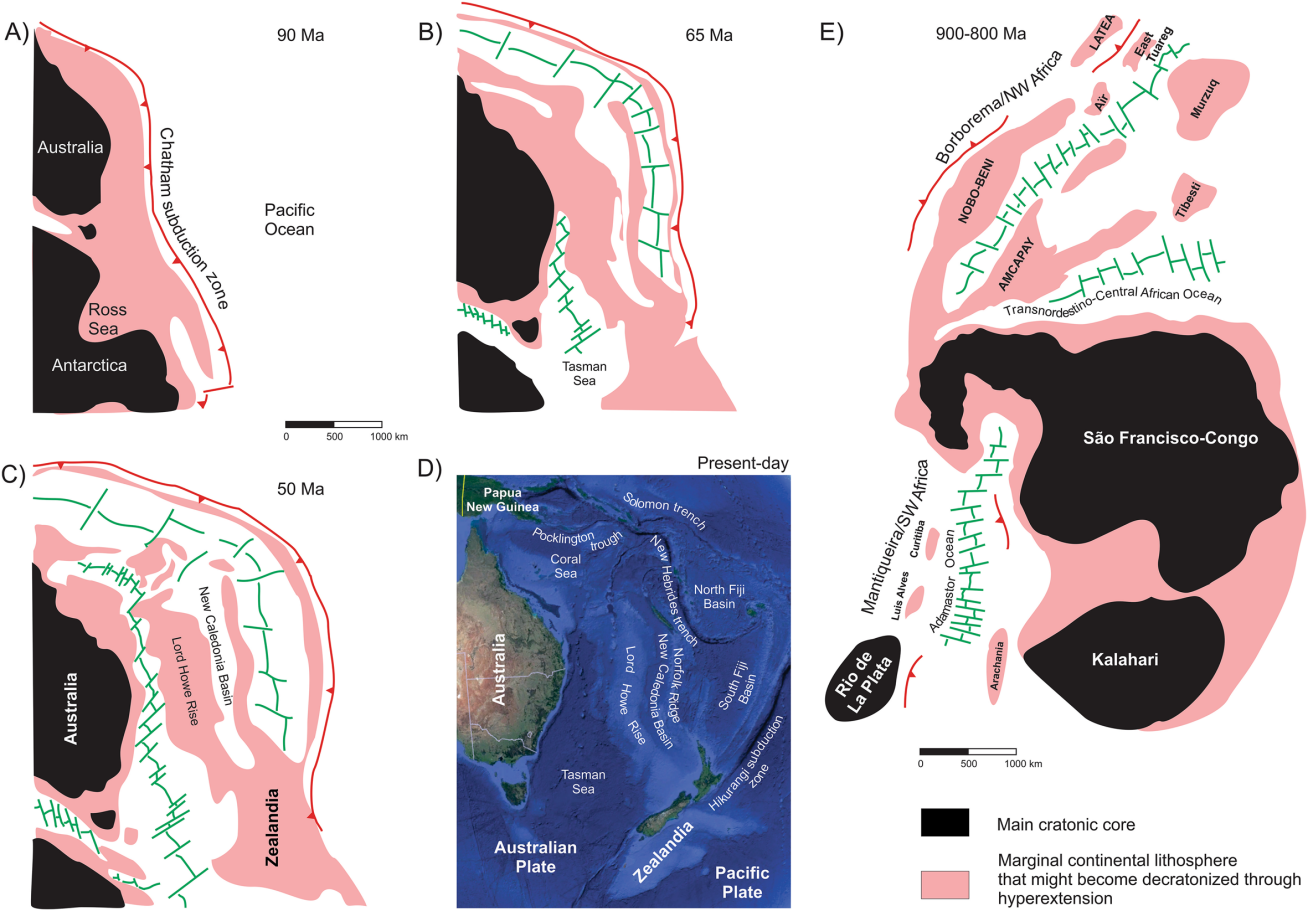
Comparison of the process of formation of the Zealandia ribbon continents (e.g. Lord Howe Rise) from hyperextension off the eastern Australian coast ((**A**), (**B**), (**C**); redrawn from^99^ after^103^; (**D**), present-day Zealandia on a Google Earth Landsat/Copernicus image) and the formation of the Mantiqueira/SW Africa and Borborema/NW Africa ribbon continents after hyperextension of a former Central African Block (**E**). Ribbon continents in Borborema named after^5,83^, as follows: *NOBO-BENI* Northern Borborema/Benino-Nigerian, *AMCAPAY* Alto Moxotó-Rio Capibaribe-Adamawa-Yadé. Maps and drawings created using Corel Draw Graphics Suite 2018 (http://www.coreldraw.com) and a Huion Kamvas GS1331B pen display (http://www.huion.com).

Perhaps the most striking present-day examples of ribbon continents formed through hyperextension lay in the recently defined Zealandia continental mass (Fig. 4) off the eastern Australian coast^102^. Zealandia is mostly (94%) submerged as a result of isostatic balance after widespread Late Cretaceous crustal thinning followed by continental breakup. Zealandia is crosscut by V-shaped oceanic basins such as the New Caledonia through, separating major continental rises such as the Lord Howe Rise and the Norfolk Ridge. The dimensions of the Lord Howe Rise (2700 km long and over 500 km wide) are similar to that of the Sibumasu ribbon continent in Indochina (ca. 3000 km long and 500–600 km wide)^99^.

## Opening and closure of V-shaped oceans: the generation of introverted orogenic systems

The hyperextension processes that ripped off the margins of the Central African block led to the opening of Tonian-Cryogenian V-shaped oceanic basins. Crucial in this respect is the concept of non-orthogonal opening of basins and propagation of rift systems^91,104^. Most marginal basins have a scissor-shape or V-shape geometry developed in response to the propagation of a rift toward a locked zone^104^. In V-shaped basins, the widely opened oceanic sectors are connected to narrow, partially enclosed oceanic basins, which, in turn, are connected to intracontinental rift systems (aulacogens) in the tip of the V.

Closure of V-shaped oceans gives rise to introverted orogenic systems, i.e. to the development of classical Wilson cycles whereupon conjugated rifted margins are rejoined in approximately the same or close to the original position after consumption of the intervening oceanic domain^105^. During closure, the widely opened oceanic domains at the free-end of the V-shaped basins become sites of subduction, leading to the development of juvenile terranes and back-arc basins, followed by collision. The terminal portions of the V’s ^104^, on the other hand, undergo rift inversion or other types of intracontinental deformation, while the confined gulf between the former and the latter might undergo subduction followed by collision without major oceanic terrane accretion. This is exactly the case of at least two orogenic systems of western Gondwana: Borborema/NW Africa (representing the closure of the Transnordestino-Central African Ocean)^5^ and Mantiqueira/SW Africa (recording the closure of the Adamastor Ocean)^81^.

### Transnordestino-Central African ocean

**Figure 5:**
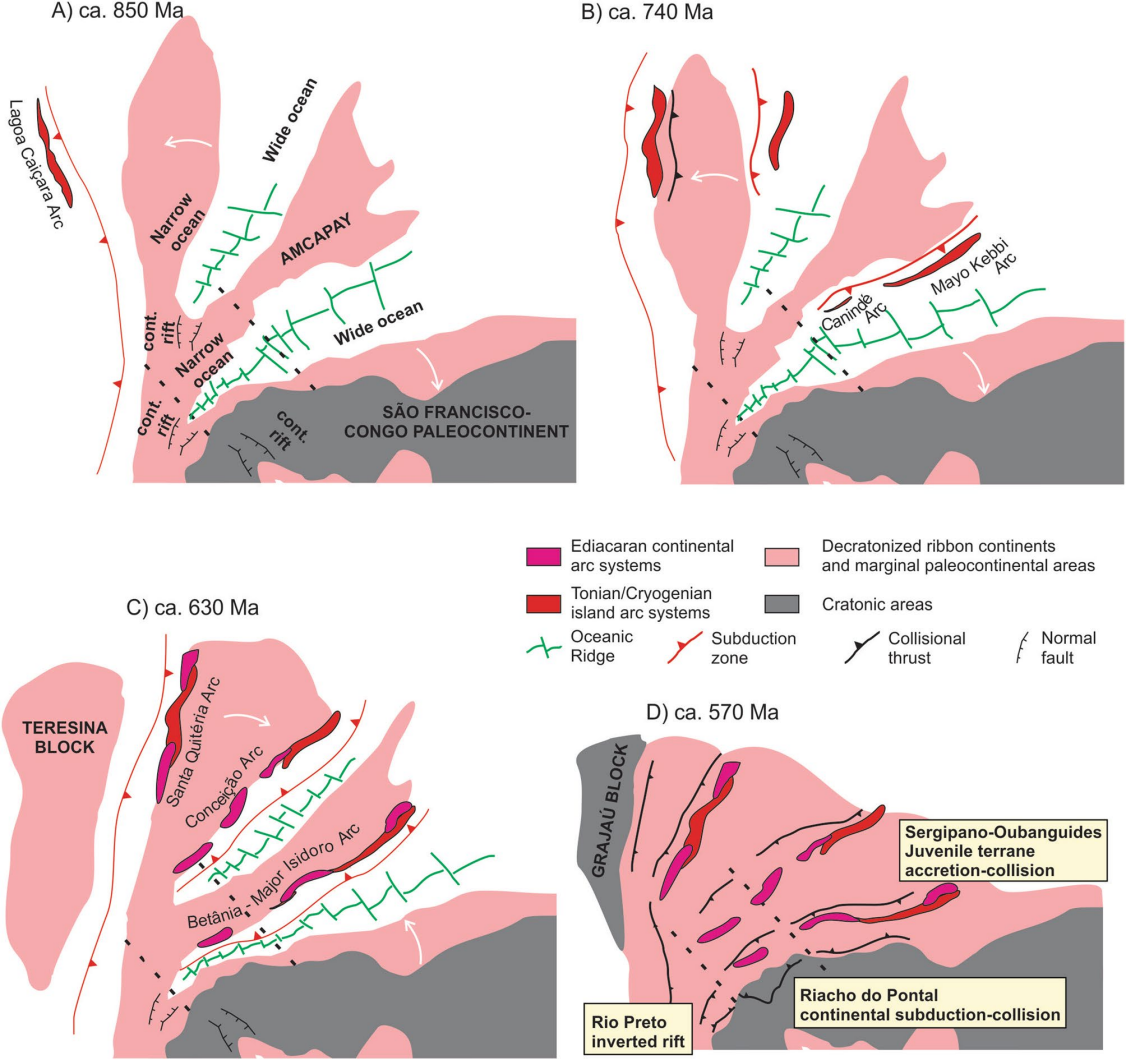
Schematic evolution of the V-shaped Transnordestino-Central African ocean in the northern São Francisco-Congo paleocontinental margin. Drawing created using Corel Draw Graphics Suite 2018 (http://www.coreldraw.com) and a Huion Kamvas GS1331B pen display (http://www.huion.com).

The northern Congo-São Francisco craton margin is marked by the curvilinear fold-thrust belts in the external zones of the Rio Preto, Riacho do Pontal, Sergipano, Yaoundé (Oubanguides), and Central African orogens^5^, which extend for more than 3000 km in the E–W direction in Brazil and Africa (Fig. 5). Together, these linked orogenic belts represent closure of the Transnordestino-Central African Ocean^5^.

The Rio Preto belt is characterized by an intracontinental and doubly-vergent deformation zone, developed during inversion of a former continental rift basin^106^. In contrast, remnants of Neoproterozoic oceanic lithosphere were found in the Riacho do Pontal Orogen (Monte Orebe ophiolite)^79^, in the Sergipano Orogen, and in the Yaoundé domain of Cameroon^80^. Cryogenian arc systems developed prior to continental collision, absent in the Riacho do Pontal Orogen, have been described in the Oubanguides^107^ and Sergipano^108^ orogens.

The Riacho do Pontal Orogen evolved through a typical Wilson Cycle^109^, preserving markers of the rift-drift-subduction-collision phases, but lacking any important intra-oceanic accretionary arc system. Its generation is regarded as due to collision of previously conjugated continental margins, respectively the northern São Francisco-Congo margin and the southern margin of the Pernambuco-Alagoas (PEAL) terrane of the Borborema Province^109^. These margins were separated at 900–880 Ma during the emplacement of a mantle plume, recorded by the Brejo Seco Ni-Cu-PGE mineralized mafic–ultramafic complex^68^, and the Paulistana rift-related metabasalts ^109^. Further east within the Borborema Province, intrusion of A-type granites at ca. 860 Ma also testify to this rifting phase^69^. Subduction and consumption of the oceanic lithosphere is marked by Ediacaran (ca. 630 Ma) continental-margin arc intrusions (Betânia Arc) in the upper plate (PEAL terrane)^110^.

Subduction and closure of the Transnordestino-Central African ocean in the Sergipano Orogen started as early as ca. 740 Ma and extended up to 680 Ma (Canindé Arc), with arc-related magmatism in a tectonic scenario similar to Ryukyu-Okinawa (Japan) and Kamchatka arc-back-arc (Russia) settings^108^. With continued subduction and consumption of oceanic crust, the arc terranes docked in the continental margins and were superseded by Ediacaran continental magmatic arc systems at ca. 630–600 Ma, which are recorded by tonalitic to granodioritic calc-alkaline magnesian plutonism^83,110–112^. Similarly, a Cryogenian arc system is interpreted in the Mayo Kebbi area of Chad/Cameroon^107^, superseded by Ediacaran plutons interpreted as continental arc intrusions^113^.

A reconstruction of the Transnordestino-Central African Ocean reveals its V-shaped configuration^104^ (Fig. 5). It is represented by an inverted rift in the V termination (Rio Preto belt), which passes eastward to a subduction-collision orogen that lacks pre-collisional intra-oceanic arc-related magmatism, but preserves remnants of an Ediacaran continental-margin arc attesting for its subduction-collision nature (Riacho do Pontal Orogen^5^) and ends with orogenic belts involving tracts of Cryogenian intra-oceanic arcs developed prior to the Ediacaran continental-margin arc systems (Sergipano and West Cameroon). Opening of this V-shaped ocean occurred through rifting and decratonization of strips of the northern Central African Block^5,31^. Akin to its opening, closure of the V-shaped ocean was also diachronous, with rotational movements of the ribbon continents back towards the cratonic margins^5,31,83^. This is evidenced by age differences of the leucocratic syn-collisional granites in the Riacho do Pontal Orogen, at ca. 610 Ma^109^ in comparison to the Sergipano Orogen, at ca. 580 Ma^111^.

### Adamastor ocean

**Figure 6:**
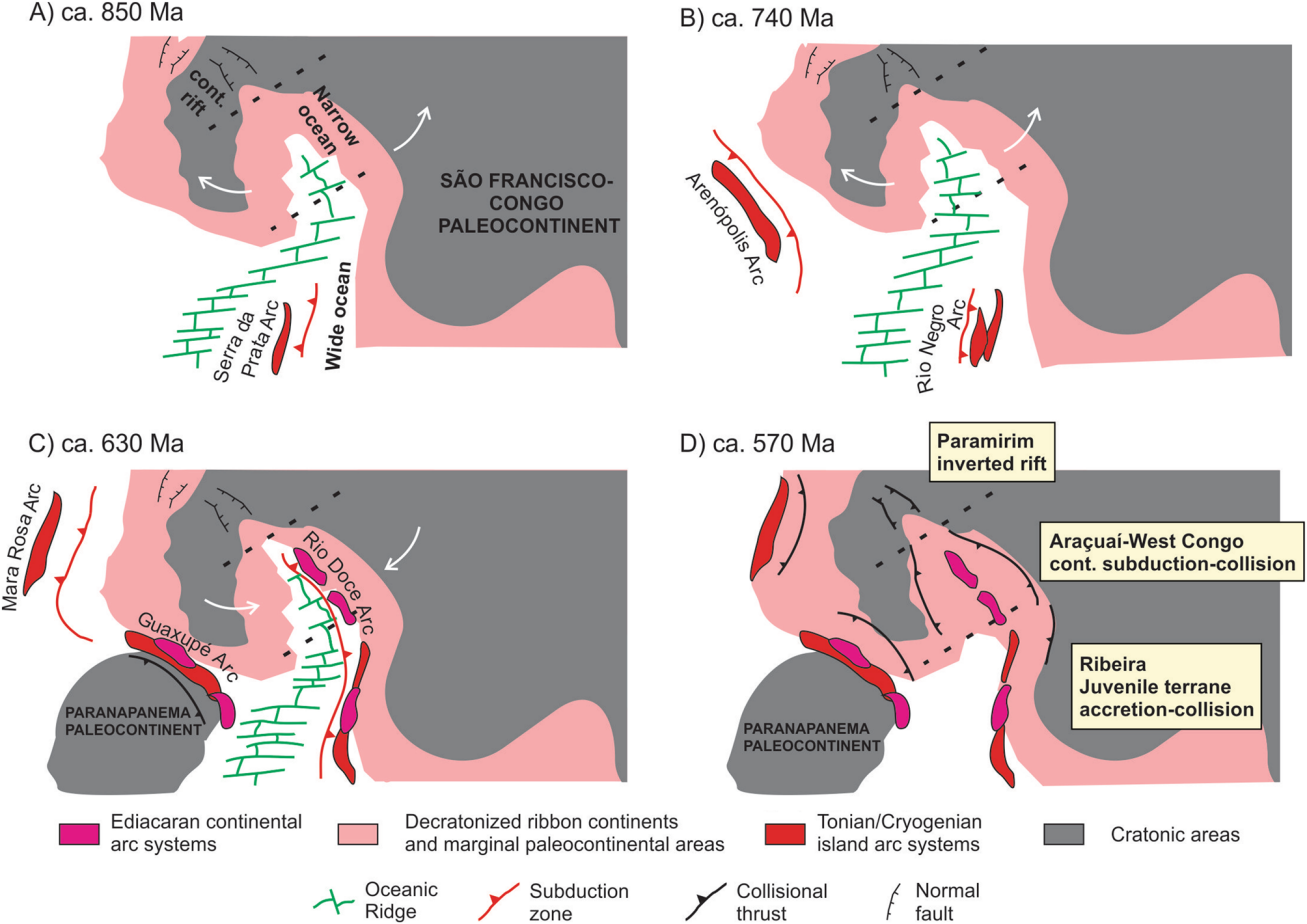
Schematic evolution of the V-shaped Adamastor Ocean in the northern São Francisco-Congo paleocontinental margin. Drawing created using Corel Draw Graphics Suite 2018 (http://www.coreldraw.com) and a Huion Kamvas GS1331B pen display (http://www.huion.com).

The Adamastor Ocean^114^ is the precursor oceanic basin to the Ediacaran/Cambrian orogenic systems of the southeastern South American Atlantic coast and its counterparts in southwestern Africa. Existence of oceanic realms in this area is recorded by accretionary mélanges containing dismembered and variably preserved ophiolite remnants located in the footwall of major shear zones interpreted as suture zones^81,115^. Evolution of the Adamastor ocean from a previous V-shaped basin was thoroughly discussed recently^81^ and evidence supporting this interpretation will not be presented here; we refer the readers to this paper for field, petrographic, geophysical, geochemical, structural and geochronological aspects of this system, combined with a discussion about its geotectonic evolution. In sum, a typical V-shaped configuration was also described for this domain, with connection of the Paramirim aulacogen in the V-shaped tip to the confined Araçuaí-West Congo Orogen (northern Mantiqueira), which in turn is connected to the Ribeira and Dom Feliciano orogens containing complex accretionary systems (Fig. 6). Of course, asinchroneity of extensional, accretionary, collisional and post-collisional activity is the rule in the thousands-of-km orogenic zone, with evidence for some areas preserving older collisional events between isolated blocks, especially in the southern orogenic regions, while other areas were undergoing accretionary or even extensional processes^81^.

## Closure of the Goiás-Pharusian ocean: generation of a major extroverted orogenic system

In contrast to the striking similarities between the São Francisco-Congo craton and reworked basement tracts in the surrounding orogenic areas (Fig. 2), the Amazonian craton presents a vastly distinct evolution. In the latter, Archean nuclei are bounded by progressively younger orogenic belts towards the SW, culminating in the collisional Sunsás Orogen at ca. 1.2–1.0 Ga^116^. Accordingly, the Amazonian craton is normally interpreted as a major fragment of Rodinia^117^.

The existence of a large Brasilides or Goiás-Pharusian Ocean separating the Amazonian paleocontinent from the São Francisco-Congo during the Neoproterozoic is well documented and testified by Tonian-Cryogenian ophiolite remnants in the Tocantins Province of central Brazil^118^ and in northeastern Brazil^119^, along with possible remnants in the Tuareg Shield of NW Africa^5^. Closure of the Goiás–Pharusian ocean occurred through crustal accretion processes, including the docking of various intraoceanic or transitional magmatic arc terranes composed of gabbro-tonalite-diorite-granodiorite-granite intrusions associated with volcanosedimentary successions bearing basalt, dacite, andesite and rhyolite volcanics and volcaniclastics, composing expanded metaluminous calc-alkaline magnesian series. These rocks date back as early as ca. 900–800 Ma and extend to ca. 680 Ma. From south to north, they are represented by:

- The early intraoceanic phase of the Goiás Magmatic Arc in the Brasília Orogen, encompassing the Arenópolis and Mara Rosa arcs^120,121^,
- The Lagoa Caiçara Arc in NW Borborema^122^,
- The early stages of the Silet (former Iskel) Arc in the Hoggar region of the Tuareg Shield^123^,
- The Amalaoulaou Arc of Mali (onset at ca. 790 Ma^124^),
- The Tilemsi Arc (ca. 730 Ma) of western Hoggar^125^,
- Arc systems exposed in the Bou Azzer and Sirwa inliers of the Anti-Atlas belt in Morocco (ca. 760–640 Ma^126^).

Whenever available, Nd, Hf, Sr and O isotope data for these massifs suggest the involvement of large volumes of juvenile mantle material^120,122,124^ in their genesis. After docking of the island and transitional arcs on the paleocontinental margins, Andean-type magmatic arcs developed, with mantle wedge melts mixing with partially melted portions of the accreted island arc together with ancient continental margin terranes, generating mixed Hf, Nd, Sr and O signatures. Continental-margin arc magmatism thus ensued in the westernmost Borborema Province at ca. 640 Ma^122^, as well as the second peak of arc magmatism in the Tocantins Province at the same time frame^121^. Diachronous continental collision ensued with collision of the Paranapanema plate with the southwestern tip of the São Francisco-Congo paleocontinent at ca. 630–600 Ma^127,128^, granulitic magmatism at ca. 570–580 Ma in the northern part of the Tocantins Province^129^ and final collision of the Amazonian paleocontinent to the amassed western Gondwana around 540–520 Ma^130^.

## Closure of the extroverted Goiás-Pharusian ocean induced closure of the introverted V-shaped Nordestino-Central African and Adamastor oceans

**Figure 7:**
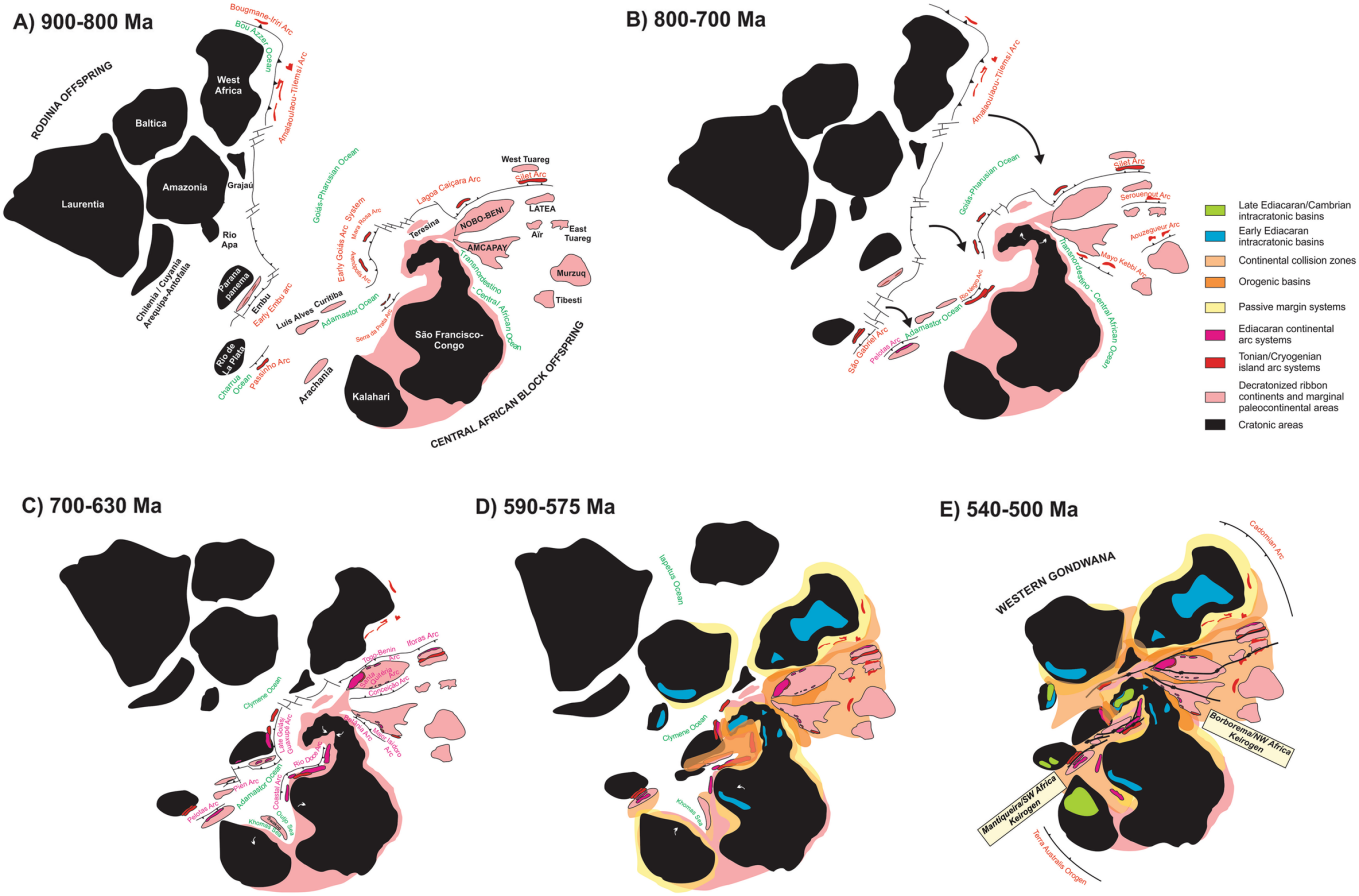
Evolutionary model for the formation of Western Gondwana. See text for explanations. Drawing created using Corel Draw Graphics Suite 2018 (http://www.coreldraw.com) and a Huion Kamvas GS1331B pen display (http://www.huion.com).

In our model (Fig. 7), the decratonized ribbon continents, microcontinents and other continental fragments that were detached from the Central African Block started drifting in windshield-wiper style towards the external oceanic domains during the Tonian/Cryogenian. This movement was, however, cut short due to subduction and ultimately closure of the Goiás-Pharusian Ocean, which led to approximation of the fragments of Rodinia (e.g. Amazonian-West African and Paranapanema paleocontinents, as well as smaller, decratonized fragments such as the Embu terrane^84,85^). Those larger cratonic fragments would ultimately collide with the ribbon continents and push them back towards the São Francisco-Congo cratonic core, the last standing rigid remnant of the Central African Block, thus characterizing the internal introverted Wilson Cycle tectonics and closure of the V-shaped oceans back in approximately the same positions, i.e. collision of formerly conjugated margins.

As the V-shaped oceans started to close, subduction of their oceanic lithosphere ensued, leading to development of late Cryogenian to early Ediacaran continental-margin magmatic arcs emplaced in the decratonized ribbon continents that acted as the upper plate in the subduction systems (Fig. 3). When the oceanic domains between these ribbon continents and the major cratonic areas were totally consumed, continental collision followed in the late Ediacaran. Due to their decratonized nature, the ribbon continents were pushed back and squeezed in between the major cratonic fragments, leading to widespread deformation and metamorphism of their weakened Archean and Proterozoic continental lithosphere.

Ultimately, contractional deformation was not able to fully accommodate the collisional stresses. Continental extrusion and lateral escape tectonics ensued, leading to the development of continental-scale networks of transpressional and transtensional shear zones, characterizing thus typical keirogens^88^ (Figs. 3 and 7), in association with post-collisional metamorphism and plutonism, mainly in the late Ediacaran and early Cambrian. The thousands-of-km long networks of shear zones that crosscut the Borborema/NW Africa and Mantiqueira/SW Africa orogenic regions were developed due to the nature of the basement in these areas, composed of various decratonized ribbon continents separated by accretionary arc systems and relics of oceanic domains. All of these tectonic units are characterized by their highly deformable nature, in contrast with the surrounding rigid cratonic cores. Thus, the major directional shear zones developed in the lateral escape phase of the Borborema/NW Africa and Mantiqueira/SW Africa orogenic systems show highly continuous linear traces for thousands of km, at places following the sites of ancient suture zones, but mostly crosscutting through and displacing virtually every geological unit in their path, regardless of age or tectonic setting, as long as those are located in the weakened decratonized or in the accretionary domains of the orogenic regions.

## Conclusions

Our model reconciles two main traits of western Gondwana that confused previous attempts of untangling its geodynamical evolution, i.e. the presence of large tracts of reworked basement within the orogenic areas and the development of large shear zone systems (keirogens) during the final stages of continental amalgamation. The main idea presented here is that Tonian hyperextension of a former Central African block, comprising what would later become the São Francisco-Congo Craton and most of the reworked basement of the Borborema/NW Africa, Tocantins and Mantiqueira/SW Africa provinces, generated a number of ribbon continents that lost their cratonic keels, were detached from the mainland, and drifted away. The rifting process generated major V-shaped oceanic basins that separated the decratonized ribbon continents from the cratonic landmass, delineating the São Francisco-Congo paleocontinental margins.

Collision of continental blocks derived from Rodinia beginning at the late Cryogenian then forced the drifting ribbon continents back towards this remainder rigid cratonic core. Subduction of the V-shaped Transnordestino-Central African and Adamastor oceanic basins and ultimately collision of the involved continental blocks generated two large orogenic systems, the Borborema/NW Africa and Mantiqueira/SW Africa, respectively. Both comprise inverted continental rifts connected to subduction-collision orogens developed from confined oceanic gulfs that in turn were connected to complex orogens involving pre-collisional accretionary systems developed in the free end of the V-shaped basins.

Collision of the Amazonian paleocontinent and other fragments of Rodinia during the late Ediacaran/Cambrian led to the final amalgamation of Gondwana. The decratonized ribbon continents became squeezed and entrained in the orogenic regions and were highly deformed, metamorphosed and intruded by syn- and post-collisional plutons. In order to accommodate the intense tangential and rotational orogenic deformation, continental extrusion and lateral escape tectonics ensued, through the development of giant networks of trans-continental shear zones both in the Borborema/NW Africa and Mantiqueira/SW Africa provinces, characterizing major keirogens (Fig. 7). The development of these shear zones systems was made possible by the highly deformable nature of the reworked ribbon continents, both due to their genesis as decratonized hyperextended basement tracts and to their former involvement in accretionary and collisional systems preserved in the interleaving former oceanic domains.

## Data Availability

All data used in this paper will be made available from the corresponding author upon reasonable request.
